# Experience of patients diagnosed as asymptomatic COVID-19 after dental treatment

**DOI:** 10.1186/s40902-021-00316-4

**Published:** 2021-09-01

**Authors:** Ju-Yeon Seo, Sung-Tak Lee, So-Young Choi, Jin-Wook Kim, Tae-Geon Kwon

**Affiliations:** Department of Oral and Maxillofacial Surgery, School of Dentistry, Kyungpook National University, 2177 Dalgubeol-daero, Jung-gu, Daegu, 41940 Republic of Korea

**Keywords:** COVID-19, SARS-CoV-2, Asymptomatic, Dental treatment, Personal protective equipment

## Abstract

**Background:**

The potential risk of coronavirus disease 2019 (COVID-19) transmission from asymptomatic COVID-19 patients is a concern in dental practice. However, the impact of this risk is not well documented to date. This report describes our dental clinical experience with patients who did not exhibit symptoms of COVID-19 but were later confirmed as positive for COVID-19.

**Case presentation:**

Of the 149,149 patients who visited the outpatient clinic of KNUDH and the 3291 patients who visited the Oral and Maxillofacial Surgery Clinic of KNUH, 3 were later confirmed as having COVID-1 between 1 February 2020 and 28 February 2021. Owing to close contact with these patients during their treatments, 46 dental and medical staff had to undergo quarantine from the date of the patients’ confirmation of COVID-19 infection.

**Conclusion:**

The presented cases showed the potential existence of asymptomatic COVID-19 patients after dental treatment with aerosol-generating procedures. Clinicians should be aware of the infection prevention measures and try to protect healthcare personnel from secondary infection of COVID-19 during dental treatments.

## Background

Severe acute respiratory syndrome coronavirus 2 (SARS-CoV-2) caused an outbreak in China in December 2019. On 11 February 2020, the disease was officially named coronavirus disease 2019 (COVID-19) by the World Health Organization (WHO). In March 2020, the crisis was declared a pandemic [1].

Transmission of SARS-CoV-2 can occur directly via inhalation of and exposure to infected secretions, such as saliva and respiratory droplets or aerosol particles, or indirectly through contact with a contaminated surface or with infected individuals [2, 3]. Close contact is defined as being within 1 m of an infected person (i.e., with laboratory-confirmed or probable COVID-19) for a total of ≥15 min over a 24-h period [4].

Studies have reported that symptomatic, presymptomatic, and asymptomatic patients can transmit SARS-CoV-2 to others [2–4]. Symptomatic transmission refers to transmission of the virus from a person showing such symptoms as fever or chills, cough, shortness of breath or difficulty in breathing, fatigue, muscle or body ache, headache, loss of taste or smell, sore throat, congested or runny nose, nausea or vomiting, and diarrhea [2, 4]. Presymptomatic transmission, which refers to the incubation period for SARS-CoV-2, is the time between exposure to the virus and the onset of symptoms (range, 2–14 days). Asymptomatic transmission refers to the transmission of the virus from a person who does not develop any symptoms [2]. In a literature review, Oran and Topol [5] reported the prevalence of asymptomatic COVID-19 to range from 40 to 45%, whereas a recent study from Korea showed that the prevalence of initially asymptomatic COVID-19 upon admission was 25.8% [6].

Dental treatments include a number of aerosol-generating procedures (AGPs), such as those that require ultrasonic scalers, air-water syringes, and air turbine handpieces, for which the possibility of airborne transmission of SARS-CoV-2 in dental practice cannot be ruled out. Virus particles with infective potential can be present in saliva and asymptomatic individuals can possibly transmit the infection. Given the high percentage of asymptomatic carriers of SARS-CoV-2, the possibility of cross-infection during dental practice cannot be excluded [7]. However, data assessing the risk of COVID-19 infection during dental clinical treatment are limited at best [8].

In this case report, we describe the circumstances surrounding contact that transpired between asymptomatic COVID-19 patients and healthcare personnel (HCP) during dental practice. The objectives of this study were to share our experience in managing the situation and to determine the necessary precautions for dental treatments involving asymptomatic COVID-19 patients.

## Case presentation

Patients who visited the Kyungpook National University Dental Hospital (KNUDH) and Kyungpook National University Hospital (KNUH) between 1 February 2020 and 28 February 2021 and were later confirmed to be infected with COVID-19 were evaluated in this study. Of the 149,149 patients who visited the outpatient clinic of KNUDH and the 3291 patients who visited the Oral and Maxillofacial Surgery Clinic of KNUH, 3 were later confirmed as having COVID-19 (Tables 1 and 2). This study was approved by the institutional review boards of the authors’ affiliated hospital (approval no. KNUH 2021-06-014, KNUDH 2021-06-03-00).

**Table 1:**
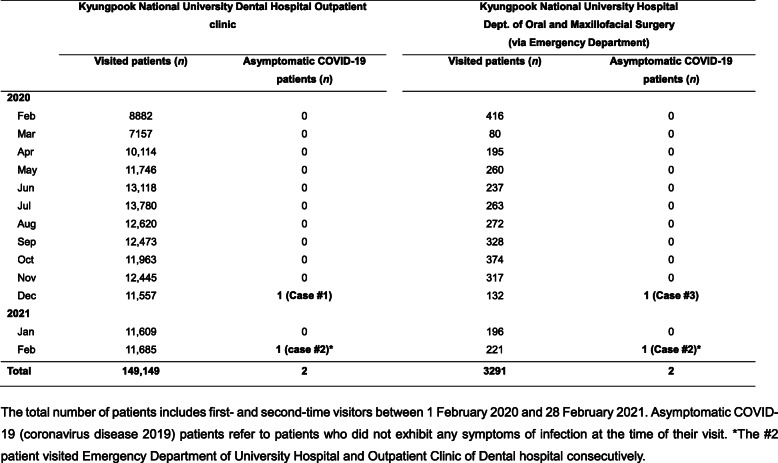
Distribution of the study population and eligible case patients

**Table 2:** Summary of dental treatments performed on asymptomatic COVID-19 patients (*n* = 3)

Case	Patient information					Staff who have been in close contact with COVID-19 patients			
	Sex/age (yr)	Hospital visit date // COVID-19 infection confirmation date	Diagnosis and treatment	Events before COVID-19 infection confirmation	Quarantine	Classification	COVID-19 test result	Quarantine period	Personal protective equipment
**1**	M/61	12/18/2020 (OPD) // 12/24/2020 (confirmed)	Chronic periodontitis; scaling (ultrasonic scaler)	12/20/2020: Attended the same worship service as a COVID-19–infected person	After 14 days of self-quarantine, confirmed negative for COVID-19	Doctor, *n* = 1	Negative	12/24/2020 to 01/01/2021	Surgical mask and latex gloves
						Dental hygienist students, *n* = 5	Negative		
**2**	F/73	02/01/2021 (ED); 02/02/2021 (OPD) // 02/12/2021 (confirmed)	Dislocation of both TMJs; TMJ reduction	A caregiver tested positive for COVID-19, and the patient had fever	Hospitalized and treated until COVID-19 test came out negative	ED Doctors, *n* = 4 Radiographers, *n* = 2	Negative	None	Surgical mask and latex gloves
						OPD Doctor, *n* = 1	Negative		
**3**	M/63	12/15/2020 (ED) // 12/16/2020 (confirmed)	Tooth injury; tooth extraction (using a high-speed handpiece) Mandibular symphysis and fracture of both condyles; IMF screw placement	After moving from the ED to the hospital ward, underwent COVID-19 test to prepare for surgery under general anesthesia	Isolated and treated in the negative pressure room at the authors’ University Hospital before being discharged	Doctors, *n* = 4	Negative	12/15/2020 to 12/29/2020	Surgical mask and latex gloves
						Nurses, *n* = 3	Negative		
						Student nurses, *n* = 2	Negative		
						Medical assistants, *n* = 3	Negative		
						Doctors, *n* = 7	Negative	12/16/2020 to 12/30/2020	
						Nurses, *n* = 11	Negative		
						Dental hygienist, *n* = 1	Negative		
						Nurses, *n* = 2	Negative	12/17/2020 to 12/31/2020	

*COVID-19* coronavirus disease 2019, *OPD* outpatient department, *ED* emergency department, *TMJ* temporomandibular joint, *IMF* intermaxillary fixation

### Case 1

A 61-year-old man visited KNUDH on 18 December 2020. The patient was diagnosed with chronic periodontitis and underwent ultrasonic scaling. On 20 December 2020, the patient attended a Sunday worship with a church member who was later confirmed to be positive for COVID-19. On 24 December 2020, reverse transcription–polymerase chain reaction (RT-PCR) confirmed that the patient was positive for COVID-19. The patient did not experience symptoms before the positive confirmation and tested negative after 14 days of quarantine.

At the time of the patient’s hospital visit, 1 doctor and 5 student hygienists participated in his treatment; they were wearing a surgical mask and latex gloves during the treatment. These 6 staff who had close contact with the patient underwent quarantine from 24 December 2020 to 1 January 2021, and tests performed before the quarantine period ended confirmed that all of them were negative for COVID-19.

### Case 2

A 73-year-old woman visited our emergency department (ED) for temporomandibular joint (TMJ) dislocation and underwent TMJ reduction on 1 February 2021. As the TMJ reduction was not successful, the patient visited our dental hospital the next day. The patient was asymptomatic to COVID-19 at the time of her visits and treatment. Ten days later, the patient was diagnosed with COVID-19 at the nursing home where the patient was a resident of. A caregiver at the nursing home initially tested positive for COVID-19. Immediately before her COVID-19 infection was confirmed, the patient had fever and was subsequently admitted to KNUH.

At the time of the patient’s visit to the ED, a doctor participated in the treatment. Because 14 days had already passed since the date of the doctor’s initial contact with the patient and the date of the patient’s confirmation of COVID-19 infection, the doctor did not undergo quarantine after testing negative for COVID-19. At the dental hospital, 4 doctors and 2 radiological technologists had close contact with the patient for the treatment. These 6 dental staff also did not undergo quarantine after confirming a negative result for COVID-19 because 13 days had passed since the date of their initial contact with the patient. All the HCP were wearing a surgical mask and latex gloves as personal protective equipment (PPE) during the treatments.

### Case 3

A 63-year-old man visited the ED of KNUH for mandibular symphysis, fracture of both condyles, and multiple teeth injury, which were caused by a slip accident on a street, on 15 December 2020. Intraoral bleeding continued owing to a number of broken teeth in the patient’s oral cavity; extraction of fractured teeth was performed using a high-speed handpiece, and intermaxillary fixation screws were placed in the ED. The patient was admitted to the ward after visiting the ED. Although the patient did not exhibit symptoms of COVID-19 infection, the patient underwent a COVID-19 test on 16 December 2020. RT-PCR result confirmed that the patient was positive for COVID-19. The patient was isolated and treated at the negative pressure room of KNUH before being discharged.

A total of 33 dental and medical staff, including 11 doctors, 16 nurses, 1 hygienist, 2 student nurses, and 3 medical assistants, were classified as close contacts of the patient. All the HCP were wearing a surgical mask and latex gloves as PPE during the treatments. As of the date of their last contact with the patient, the 33 staff underwent 14 days of quarantine, and all of them were COVID-19 negative based on laboratory tests performed before their release from quarantine.

## Discussion

This report describes the cases of 3 asymptomatic patients who visited our hospital for elective and emergency dental treatments and were later confirmed as having COVID-19 infection. A total of 46 dental and medical staff who were in close contact with these patients during their treatments had to undergo quarantine from the date of the patients’ confirmation of COVID-19 infection. None of the hospital staff tested positive for COVID-19 after coming in close contact with the asymptomatic COVID-19 patients, and no further cases had been linked to the patients. During the same period as this study, 8623, 3255, and 89,676 COVID-19–positive patients were identified in Daegu Metropolitan City, nearby province (Kyungpook Province), and South Korea, respectively [9].

COVID-19 infection in asymptomatic patients can be confirmed in 2 ways: One is when a public health center is notified that a COVID-19–positive person had visited the facility, and another is when a patient who visits a hospital is directly tested and confirmed as having COVID-19 infection. In both scenarios, which can happen at any time, dental treatment poses a risk to dental staff.

According to the interim guidance released by the US Centers for Disease Control (2020) [8] and WHO (2020) [10], oral healthcare workers are likely to come in close contact with patients’ faces for a prolonged time during dental procedures. Examples of close contact include face-to-face communication; frequent exposure to saliva, blood, and other body fluids; and using sharp instruments. Various dental instruments, such as high- or low-speed handpieces, an ultrasonic scaler, or a 3-way air-water spray, can generate aerosol particles, which may cause airborne transmission [3, 8, 10]. Airborne transmission is defined as the spread of an infectious agent caused by the dissemination of droplet nuclei (aerosols), which remain infectious when suspended in air over long distances and time [2, 4]. Usually, regularly performing AGPs on patients with COVID-19 and working with infected people in indoor, crowded places without adequate ventilation carry a high risk of COVID-19 transmission [2, 4]. In addition, individuals who are in close contact with patients with symptomatic or asymptomatic COVID-19, including healthcare workers and other patients in the hospital, are at a higher risk of COVID-19 infection [11].

WHO recommends the use of fluid-resistant medical masks or respirators if an AGP is anticipated, fluid-resistant gowns, gloves, and eye protection (goggles or face shield) as PPE for dental surgery as well as performing proper hand hygiene. A fit-tested N95 or FFP2 respirator (or higher) is recommended when AGPs are performed [3, 8, 10]. Although many guidelines or reports have suggested the high possibility of transmission of COVID-19 via dental procedures [7], cases of COVID-19 transmission from such procedures have not been frequently reported. In this study, we also did not find a case of SARS-CoV-2 transmission during any of the dental treatments.

A study on the prevalence of COVID-19 among dentists in the USA found that 20 of 355 tested dentists were diagnosed as having confirmed or probable COVID-19 infection [11]. The probable sources of virus transmission were determined via contact tracing; notably, dental procedures were not identified as a source of transmission among the reported cases. A report from Italy also indicated that transmission of SARS-CoV-2 between patients and dentists or dental assistants was not found after treatment in a dental hospital [12]. In a recent case report, even in the presence of SARS-CoV-2 from a throat swab, SARS-CoV-2 RNA was not detected from the oral mucosal tissue; therefore, the true risk of COVID-19 infection from oral tissues cannot be confirmed [13]. A recent paper on the origin of microbes of aerosol that occurred during dental treatment showed that SARS-CoV-2 was detected in the saliva of asymptomatic COVID-19 patients, but not in the aerosol [14]. However, it is needed to be emphasized that saliva is known to contain SARS-CoV-2 RNA and the oral cavity is regarded as the main infection transmission route [15]. Therefore, these results suggest that the risk of SARS-CoV-2 transmission from aerosolized saliva during dental treatment needs to be investigated. A prospective study with a large number of patients should be conducted in the future.

The incubation period, which is the time between exposure to the virus and symptom onset, for COVID-19 is, on average, 5 to 6 days, but it can take as long as 14 days [2–4]. Early data from China suggested that people without symptoms of COVID-19 could infect others [16]. Distinguishing between transmission from people who are infected but never develop symptoms (asymptomatic transmission) and transmission from people who are infected but have not developed symptoms yet (presymptomatic transmission) is crucial in better understanding the role of transmission from infected people without symptoms [7, 8, 10].

From our experience of treating COVID-19–positive patients who were asymptomatic, additional infections did not occur among dental and medical staff. Practitioners should keep abreast of new scientific knowledge and protection guidelines regarding COVID-19 because they could be treating asymptomatic patients at any given time. Further research, including investigation into the distinction between asymptomatic infection and presymptomatic transmission, is needed to be continued.

## Conclusion

The current report of cases showed the potential existence of asymptomatic COVID-19 patients after dental treatment with aerosol-generating procedures. Clinicians need to be fully aware of the infection prevention measures and try to protect healthcare personnel from secondary infection of COVID-19.

## Data Availability

Not applicable. (Data sharing is not applicable to this article as datasets were not generated or analyzed during the study.)

## Abbreviations

COVID-19: Coronavirus disease 2019
SARS-CoV-2: Severe acute respiratory syndrome coronavirus 2
WHO: World Health Organization
AGPs: Aerosol-generating procedures
HCP: Healthcare personnel
RT-PCR: Reverse transcription–polymerase chain reaction
ED: Emergency department
TMJ: Temporomandibular joint
PPE: Personal protective equipment
CT: Computed tomography
